# Cost Effectiveness Analysis of Clinically Driven versus Routine Laboratory Monitoring of Antiretroviral Therapy in Uganda and Zimbabwe

**DOI:** 10.1371/journal.pone.0033672

**Published:** 2012-04-24

**Authors:** Antonieta Medina Lara, Jesse Kigozi, Jovita Amurwon, Lazarus Muchabaiwa, Barbara Nyanzi Wakaholi, Ruben E. Mujica Mota, A. Sarah Walker, Ronnie Kasirye, Francis Ssali, Andrew Reid, Heiner Grosskurth, Abdel G. Babiker, Cissy Kityo, Elly Katabira, Paula Munderi, Peter Mugyenyi, James Hakim, Janet Darbyshire, Diana M. Gibb, Charles F. Gilks, on behalf of the DART Trial Team

**Affiliations:** 1 Health Economics Group, Peninsula College of Medicine and Dentistry, Exeter University, Exeter, United Kingdom; 2 Joint Clinical Research Centre, Kampala, Uganda; 3 Medical Research Council/Uganda Virus Research Institute Research Unit on AIDS, Entebbe, Uganda; 4 University of Zimbabwe Clinical Research Centre, Harare, Zimbabwe; 5 Medical Research Council Clinical Trials Unit, London, United Kingdom; 6 Infectious Diseases Institute, Kampala, Uganda; 7 Faculty of Medicine, Imperial College London, London, United Kingdom; University of New South Wales, Australia

## Abstract

**Background:**

Despite funding constraints for treatment programmes in Africa, the costs and economic consequences of routine laboratory monitoring for efficacy and toxicity of antiretroviral therapy (ART) have rarely been evaluated.

**Methods:**

Cost-effectiveness analysis was conducted in the DART trial (ISRCTN13968779). Adults in Uganda/Zimbabwe starting ART were randomised to clinically-driven monitoring (CDM) or laboratory and clinical monitoring (LCM); individual patient data on healthcare resource utilisation and outcomes were valued with primary economic costs and utilities. Total costs of first/second-line ART, routine 12-weekly CD4 and biochemistry/haematology tests, additional diagnostic investigations, clinic visits, concomitant medications and hospitalisations were considered from the public healthcare sector perspective. A Markov model was used to extrapolate costs and benefits 20 years beyond the trial.

**Results:**

3316 (1660LCM;1656CDM) symptomatic, immunosuppressed ART-naive adults (median (IQR) age 37 (32,42); CD4 86 (31,139) cells/mm^3^) were followed for median 4.9 years. LCM had a mean 0.112 year (41 days) survival benefit at an additional mean cost of $765 [95%CI:685,845], translating into an adjusted incremental cost of $7386 [3277,dominated] per life-year gained and $7793 [4442,39179] per quality-adjusted life year gained. Routine toxicity tests were prominent cost-drivers and had no benefit. With 12-weekly CD4 monitoring from year 2 on ART, low-cost second-line ART, but without toxicity monitoring, CD4 test costs need to fall below $3.78 to become cost-effective (<3xper-capita GDP, following WHO benchmarks). CD4 monitoring at current costs as undertaken in DART was not cost-effective in the long-term.

**Conclusions:**

There is no rationale for routine toxicity monitoring, which did not affect outcomes and was costly. Even though beneficial, there is little justification for routine 12-weekly CD4 monitoring of ART at current test costs in low-income African countries. CD4 monitoring, restricted to the second year on ART onwards, could be cost-effective with lower cost second-line therapy and development of a cheaper, ideally point-of-care, CD4 test.

## Introduction

It is essential to evaluate the economic impact of antiretroviral therapy (ART) programmes using a public health approach [1], to guide policymakers how best to prioritise scarce resources for HIV/AIDS care and treatment in the public sector. This is particularly important with the current financial crisis, threats to sustained HIV programme funding and the many individuals still in urgent need of first and increasingly second-line treatment, particularly in South and Eastern Africa [2]–[4].

Several studies have evaluated the clinical benefit [5]–[11] and cost-effectiveness [12]–[18] of different strategies for monitoring the safety and efficacy of ART. Results have been contradictory; for example, in one modelling study of ART in resource-limited settings [15], monitoring with viral loads or CD4 cell counts compared with clinical assessment alone led to only modest benefits in patient survival and drug resistance. Conversely, a recent modelling study for Côte d’Ivoire, a lower-middle income country with a GDP per capita of $1071, found both monitoring strategies cost-effective [17]. A recently published trial from Uganda concluded that, compared with clinical monitoring alone, routine CD4 count monitoring is considerably more cost effective alone than combined with viral load monitoring [18].

Here we present a cost-effectiveness analysis, from the public healthcare perspective, of Laboratory and Clinical Monitoring (LCM) compared with Clinically-Driven Monitoring (CDM) of ART alongside a randomised controlled trial conducted in Uganda and Zimbabwe [11].

## Methods

### Ethics Statement

The DART trial was approved by Research Ethics Committees in Uganda, Zimbabwe and the UK, and all enrolled participants gave individual informed consent.

Effectiveness and resource utilisation data were collected in the Development of Antiretroviral Therapy (DART) trial, conducted from 2003–2008 at 3 centres in Africa: Entebbe and Kampala (plus satellite), Uganda; Harare, Zimbabwe. The trial compared LCM (routine 12-weekly laboratory monitoring: CD4 counts for efficacy; haematology and biochemistry for toxicity) with CDM (CD4 counts never available to clinicians) in ART-naïve adults (≥18 years) starting therapy with symptomatic HIV disease and CD4<200 cells/mm^3^. Additional diagnostic investigations and monitoring tests (except CD4 in CDM) could be requested according to clinical judgement at routine visits, patient-initiated visits or hospital admission. Concomitant medicines were provided. The trial demonstrated no impact of routine toxicity tests on any adverse event outcome; and a small, statistically significant benefit of CD4 monitoring on HIV disease progression and death from the second year onwards [11]. DART also showed that regular fixed duration ART interruptions to reduce drug costs were harmful [19]; and that cotrimoxazole prophylaxis on ART significantly reduced mortality [20].

Mean total cost per patient by group was estimated using intention-to-treat. Research component costs were separated from those for ART delivery/monitoring and excluded from analysis. Individual patient data on healthcare resource utilisation and health outcomes of all DART participants were analysed, except for concomitant medications which were analysed by group. Because of the large number of different medications used over the 6 year trial, only those used by >30% of patients, or where there was difference of >3% in the proportion using a medication between the two randomised groups were accounted for. Healthcare utilisation outside of the trial (health clinic visits, hospitalisations and concomitant medications) were elicited at every 4-weekly visit and included. Unit costs of CD4 counts, biochemistry, haematology, clinic and health centre visits were estimated in primary economic costing analyses [21] for each centre, using costs from the main Kampala centre for its satellite. Staff costs per routine and extra patient-initiated visits at DART clinics were estimated in a time and motion study at the centres (patient visits: Entebbe, n = 152; Kampala, n = 171; Harare n = 175). Financial information was obtained from trial centre records. ART costs were based on preferential prices of non-generic drugs for sub-Saharan Africa in 2008 [22]. Country-specific costs for hospital bed-days were obtained from WHO [23].

All local prices were converted to 2008 US$ using exchange rates over the trial period (e.g. $1US$ = 1,900 Uganda Shillings; 1US$ = 777,500,000 Zimbabwean dollars in 2008) and the US Consumer Price Index [24]. Official daily Zimbabwean rates were applied to essential medical equipment and supplies, and market rates from independent sources to staff salaries and locally acquired items. No adjustment for purchasing power parity was attempted given the lack of comparable standards in price level information sources.

Survival benefits were estimated from the difference in the area under the Kaplan-Meier [25] survival curve between the two groups up to the trial end. Quality-adjusted life years (QALYs) were calculated as the weighted total time spent in four possible health states during the trial, with each state’s utility as its weight. Health states were defined by performance scale assessment (1 = asymptomatic; 2 = symptomatic; 3 = bed-ridden <50% in the previous month; 4 = bed-ridden >50% in the previous month) during 12-weekly routine doctor visits and currently-ongoing WHO stage 3/4 events. Hence, health states, unlike WHO staging, may improve from baseline. Utilities for estimating QALYs were obtained from a longitudinal sub-study (n = 275) conducted at the DART Entebbe centre (see Supporting Information S1), relative to the utility of the asymptomatic HIV-infected state. This state was assumed to be optimal, thus having a value of 1, to HIV-infected patients, and 0.81, to the general population [26].

Incremental cost effectiveness ratios (ICERs) defined as the ratio of incremental mean cost to mean survival or QALY gained, were estimated. Costs and health benefits occurring after 12 months were discounted at 3% per annum, and adjusted for censoring due to drop-out using the method of Li [27]. 95% confidence intervals for differences in means and ICERs were estimated using bootstrap percentile methods [28]. Sensitivity analyses included generic drug prices (WHO [29]), reduced prices for second-line therapy [22], laboratory monitoring costs from the literature [30], and National Referral Laboratories prices.

A Markov model of transitions between discrete CD4 states and death was used to extrapolate outcomes post-DART, from 6 to 25 years after ART initiation. An individual alive at trial closure would be in one of three states, defined by CD4 <100, 100–200, and >200 cells/mm^3^, qualified by first-line or second-line ART due to expected differences in costs and benefits. After every 12-week period, the participant would have moved to a different state, remained in the initial state or died. Overall 97.7% of expected CD4 counts before end of trial/death/loss to follow-up were available. For routine CD4 tests, clinic visits and ART use, full compliance with the DART follow-up schedule was assumed. Other costs and QALYs were analysed as random events, divided into 12-week periods and matched to latest CD4 count range to derive estimates of cost and benefit (‘pay-offs’) for Markov states as a function of latest CD4 count and group. In this extrapolation, only cotrimoxazole prophylaxis and treatment and antimalarial drugs, were included as concomitant medications; the former were the only concomitant medications whose use varied significantly over years on ART and the latter were the only medications whose use varied significantly across centres. The analysis accounted for background mortality risks derived from counterfactual life tables for Uganda that exclude the effect of HIV/AIDS (see Supporting Information S2).

ICERs <3x per capita GDP were used as an indication of cost-effectiveness, following benchmarks suggested by WHO [31]. Under a fixed, limited healthcare budget, an alternative threshold applies, equal to the ICER of CDM versus the ‘no ART’ option (see Supporting Information S2). The probability of cost-effectiveness over different threshold values, i.e., the cost-effectiveness acceptability curve, was derived from probabilistic sensitivity analysis.

## Results

In DART, 3316 (1660 LCM; 1656 CDM) HIV-infected, treatment-naïve, symptomatic African adults started ART (65% female; median (IQR) age 37 (32–42); CD4 86 (31–139) cells/mm^3^) and were followed for median 4.9 (4.5–5.3) years. Unit costs are presented in Table 1. The annual cost of the first-line regimens used in DART was US$309–585; switching to second-line regimens resulted in approximately three-fold higher ART costs.

**Table 1:** Unit costs in 2008 US$.

Item	Observed			Source and Date	Sensitivity Analysis	Source and Date
First line therapy annual cost*						
ZDV+3TC+TDF	$349.12			Midpoint of MSF prices, 2009 [18]	$290.72	Generic prices (from WHO)2008 [25]
ZDV+3TC+NVP	$308.79				$165.64	
ZDV+3TC+ABC	$585.46				$441.80	
Second-line therapy annual cost*						
ddI+EFV+KAL	$1032.59			Midpoint of MSF prices, 2009 [18]	$874.18	Lower bound of MSF prices,2009 [19]
ddI+NVP+KAL	$967.98				$763.22	
ddI+ABC+KAL	$1244.65				$1062.52	
CD4 cell counts	$15.79	Entebbe		Micro-costing– centre specific, 2008	$8.82	Rosen, Long and Sane,2008 [26]
	$10.07	JCRC				
	$18.82	UZCRC				
Haematology panel tests	$6.46	Entebbe			$5.30	National Referral Laboratories, 2008
	$7.13	JCRC				
	$12.32	UZCRC				
Biochemistry panel tests	$11.66	Entebbe			$29.50	
	$10.67	JCRC				
	$12.23	UZCRC				
DART visits	$4.08		Entebbe		N/A	N/A
	$3.30		JCRC			
	$8.38		UZCRC			
Health Centre visits	$3.26		Entebbe		N/A	N/A
	$8.84		JCRC			
	$8.73		UZCRC			
Per diem hospital cost†	$25.57			Adam, Evans & Murray, 2003 [17]	N/A	N/A
Other diagnostic investigations and tests‡	$6.26			National Referral Laboratories, 2008	N/A	N/A

* Annual drug costs of keeping a patient on this specific combination of drugs continuously for 365 days.

† Per diem hospital costs from Adam T, Evans DB and Murray CJL, 2003 were reflated to 2008 USD($).

‡ Other unit costs for diagnostic investigations (including X rays, TB smears, CSF analysis etc) and non-routine biochemistry and haematology tests are not listed here but were obtained from National Referral Laboratories prices list.

Healthcare resources consumed by patients, overall costs of resource utilisation and cost differences between the groups are shown in Table 2. The overall mean incremental cost of LCM is US$765 [95%CI 685,845] per patient, a 31% increase over CDM. The drivers of cost differences between LCM and CDM are, in order of magnitude, routine 12-weekly toxicity monitoring, CD4 monitoring, and second-line drugs. There are small offsets with lower costs of clinically-indicated diagnostic investigations, first-line therapy and fewer days in hospital in LCM, likely consequences of earlier switch to second-line therapy in LCM [11].

**Table 2:** Healthcare resource utilisation (US$2008).

Healthcare resource utilisation	Observed total over trial period per patient (median 4.9 years)		Cost difference LCM – CDM *
	LCM	CDM	
First-line therapyNumber of days Mean (SD)Costs Mean (SD)	1464.96 (555)1451 (603)	1481.90 (545)1470 (603)	−19
Second-line therapyNumber of days Mean (SD)Costs Mean (SD)	152.99 (355)406 (964)	102.89 (270)265 (718)	+141
CD4 monitoringNumber of CD4s Mean (SD)Costs Mean (SD)	19.81 (6)288 (121)	0 (0)0 (0)	+288
Routine 12-weekly haematology toxicity monitoringNumber of haematology tests Mean (SD)Costs Mean (SD)	21.58 (7)183 (81)	0 (0)0 (0)	+183
Routine 12-weekly biochemistry toxicity monitoringNumber of CD4s biochemistry tests Mean (SD)Costs Mean (SD)	20.93 (6)227 (69)	0 (0)0 (0)	+227
Clinically indicated diagnostic investigations, other tests outsideroutine visits (both groups) or monitoring tests (except CD4)requested for clinical reasons at routine visits (CDM)Number of diagnostic investigationsCost Mean (SD)	6.56 (11)41 (81)	10.49 (18) †66 (170)	−25
DART clinic visitsNumber of visits Mean (SD)Costs Mean (SD)	61.18 (19)414 (195)	60.12 (20)405 (197)	+9
Health centre visitsNumber of visits Mean (SD)Costs Mean (SD)	7.73 (7)53 (56)	7.88 (8)55 (64)	+2
Nights in hospitalNumber of visits Mean (SD)Costs Mean (SD)	5.51 (13)141 (347)	6.77 (16)177 (444)	−36
Concomitant medications	46	48	−2
Overall mean total costs (SD)[95% confidence interval]	3249 (1246)	2485 (1095)	765[685,845] ‡

* Discrepancies in totals and differences are due to rounding.

† 1.47 (3) and 0.86 (3) of the total were standard haematology or biochemistry tests respectively performed at routine doctor visits as part of the trial for CDM but requested for clinical management.

‡ 95% CIs were estimated with bootstrapping percentile method.

Mean survival benefit with LCM was 0.112 life-years (41 days). The unadjusted incremental cost per life year gained (LYG) was US$6819, with 95% CI from US$3282 to the dominated outcome where it produces lower survival benefits alongside higher costs than CDM (Table 3a). After adjusting for censoring due to drop-out and discounting costs and benefits occurring after year one, the ICER per LYG was $7386 [95%CI 3277,dominated]). The incremental cost per QALY gained was $7793 [4442,39179] using patients’ utility values, and $9621 [5484,48359] adjusting utilities to the general population’s values. Across DART centres, total mean costs of LCM and CDM ranged from $2647–3358 and $1930–2490 respectively, with adjusted discounted incremental costs of $8420–12340 per QALY gained adjusting utilities to the general population’s values (data not shown).

**Table 3:** Main and sensitivity analyses: life years gained, QALYs, costs and ICERs.

	LCMN = 1656	CDMN = 1660	DifferenceLCM – CDM
**(a) MAIN RESULTS**			
Overall survival days* [95% CI] (undiscounted)	2000	1959	+41 [−15,+95]
Overall survival years* [95% CI] (undiscounted)	5.48	5.36	+0.112 [−0.04,+0.26]
Overall mean total costs in US$ [95% CI] (unadjusted)	3249	2485	+765 [685,845]
Overall QALYs – patients’ values	3.351	3.255	+0.096
Overall QALYs – general population’s values	2.714	2.636	+0.078
Overall mean total costs in US$ [95% CI]	3146	2398	+748 [+679,+818]
Incremental Cost per Year Gained in US$ (unadjusted) [95% CI]	6819 [3282, Dominated]		
Incremental Cost per Life Year Gained in US$ [95% CI]	7386 [3277, Dominated]		
Incremental Cost per QALY Gained in US$ – patients’ values [95% CI]	7793 [4442,39179]		
Incremental Cost per QALY Gained – in US$ general population’s values [95% CI]	9621 [5484,48359]		
**(b) SENSITIVITY ANALYSIS**			
**Lower bound prices second-line therapy** *			
Mean second-line therapy costs in US$ (SD)	357 (845)	236 (637)	+121
Overall mean total costs in US$ (unadjusted) [95% CI]	3194	2449	+745 [669,821]
Incremental Cost per Life Year Gained in US$ (unadjusted) [95% CI]	6643 [3307, Dominated]		
Incremental Cost per Life Year Gained in US$ [95% CI]	6443 [2891, Dominated]		
Incremental Cost per QALY Gained in US$ – patients’ values [95% CI]	6798 [3917, 30501]		
Incremental Cost per QALY Gained in US$ – general population’s values [95% CI]	8392 [4836,37656]		
**National referral laboratory prices** *			
Mean CD4 monitoring costs in US$ (SD)	175 (57)	0 (0)	+175
Standard 12-weekly haematology biochemistry toxicity monitoring (SD)	699 (216)	23 (65)	+676
Overall mean total costs in US$ (unadjusted) [95% CI]	3425	2493	+932 [+851,+1013]
Incremental Cost per Life Year Gained in US$ (unadjusted) [95% CI]	8318 [3876, Dominated]		
Incremental Cost per Life Year Gained in US$ [95% CI]	8990 [4160, Dominated]		
Incremental Cost per QALY Gained in US$ – patients’ values [95% CI]	9485 [5334, 47957]		
Incremental Cost per QALY Gained in US$ – general population’s values [95% CI]	11710 [6586, 59206]		

* See column sensitivity analysis in Table 1.

In sensitivity analyses with prices for low-cost second-line drugs, ICERs reduced: the adjusted incremental cost per LYG was $6443 [2891, Dominated]; and per QALY was $6798 [3917,30501] using patients’ values and $8392 [4836,37656] using general population values (Table 3b). Generic drug prices would reduce ICERs by 0.3%, since the resulting rise in incremental LCM costs of first-line consumption is offset by reduced additional costs of second line therapy (results not shown). Incremental costs per LYG and QALY increased in the sensitivity analysis of laboratory costs because the National Referral Laboratory biochemistry panel cost considerably more than in DART, outweighing lower CD4 and haematology costs. All these results are above the (weighted average) threshold of cost-effectiveness for Uganda and Zimbabwe of $1,200 for 2008 [24].

Scenario analyses were undertaken to explore the cost-effectiveness of monitoring strategies not directly evaluated in DART. In a limited CD4 monitoring scenario without routine toxicity monitoring, 12-weekly CD4 monitoring was restricted to the second year on ART onwards (as outcomes between groups differed only from the third year on ART following differences in rates of switching to second-line ART from the second year [11]). This generated an adjusted discounted incremental cost per LYG of US$2997 [95%CI 1333,dominated], US$3162 [1815,14178] and US$3903 [2241,17504] per QALY gained based on patient and general population values respectively. If low second-line therapy costs are also assumed, the cost per CD4 count needs to fall below $3.78 to become cost-effective in this trial-based analysis.

**Figure 1:**
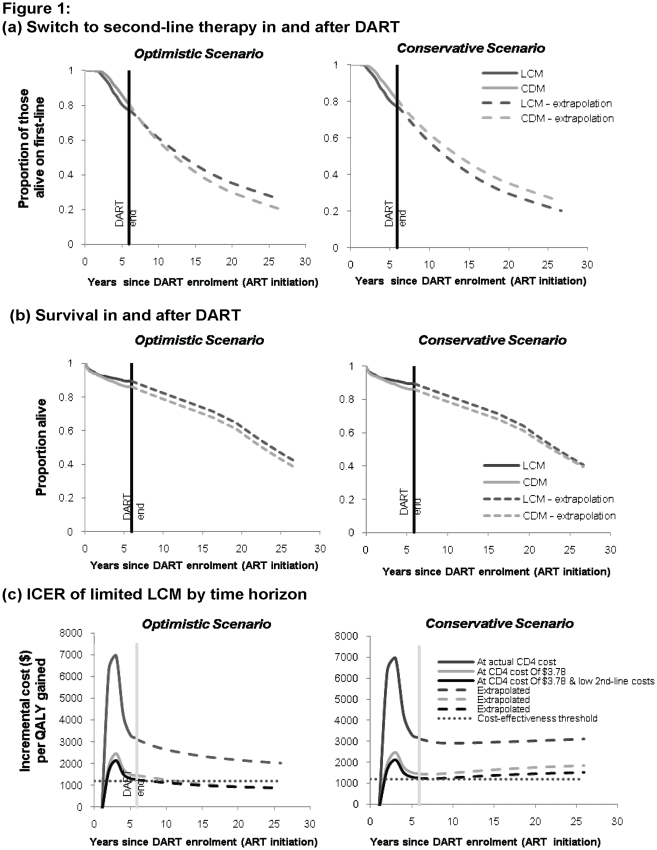
Long-term extrapolation of outcomes and ICERs.

ART outcomes were extrapolated long-term by applying predicted transition probabilities corresponding to the last two years of DART (see Supporting Information S2): at CD4 >200 cells/mm^3^, LCM has a lower switch rate than CDM, and vice versa at low (<100 cells/mm^3^) CD4 levels. In our optimistic scenario, this differential was applied; whereas in the conservative scenario both groups were assigned the same probability of switching to second-line at high CD4 counts. Long-term differences emerge: from the third year after DART onwards, a higher proportion of CDM participants would be on second-line therapy in the optimistic scenario because of the greater switch rate at high CD4 levels where most time was spent; whereas in the conservative scenario, a higher proportion of LCM participants continue to have switched to second-line (Figure 1; top panel). After 25 years of ART, 60% of the participants would have died. Only in the optimistic scenario is the survival benefit of LCM maintained (Figure 1; middle panel). This continued survival differential is due to second-line therapy having higher mortality risks than first-line, and more CDM participants on second-line therapy in the long-term optimistic scenario.

The ICER per QALY gained over time is shown for the limited laboratory monitoring (CD4 from the second year on ART only) scenario in Figure 1, bottom panels under three cost conditions: a) CD4 cost observed in DART ($14.46, weighted average across centres); b) hypothetical, CD4 test ($3.78) and c) hypothetical CD4 test and lowest observed second-line costs ($763; see Table 1). The scenarios employ patients’ utility values. In the optimistic scenario, ICERs decline with time on therapy, driven by the cumulative gap in survival and second-line therapy use between groups, although this effect would be dampened under a discount rate higher than 3% (not shown). In the optimistic scenario condition (c), the cost per CD4 count needs to fall to <$6.75 for the ICER to remain under $1200. Under a fixed budget the maximum cost-effective value is $5.81 (see Supporting Information S2).

**Figure 2:**
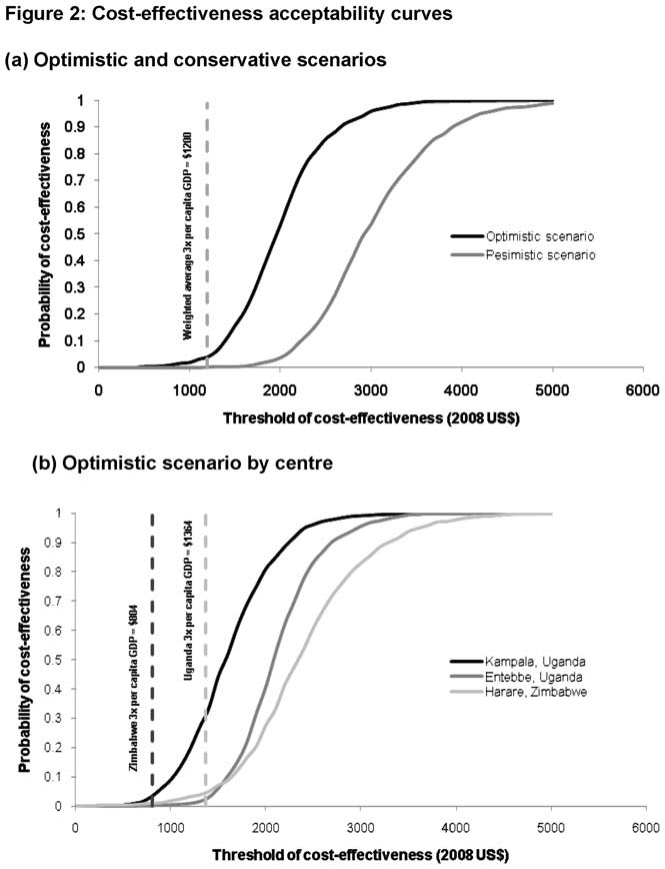
Cost-effectiveness acceptability curves.

Figure 2 shows the probability of cost-effectiveness as a function of the monetary threshold per QALY gained under condition (a) above. In the conservative scenario, there is zero probability of cost-effectiveness at the US$1200 threshold: in the optimistic scenario this probability increases to less than 5% (Figure 2a). These aggregate results mask variation between centres stemming from cost and monetary threshold differences. In the optimistic scenario, limited CD4 monitoring had a <4% probability of cost-effectiveness in Entebbe and Harare at country-specific thresholds (unit cost of CD4 count tests) of $1364 ($15.79) and $804 ($18.82) respectively, and 30% probability of cost-effectiveness in Kampala with a CD4 unit cost of $10.07 (Figure 2b). The country-specific cost-effectiveness thresholds were achieved at test costs of $7.87, $7.43, and $3.39, respectively, in the three centres over the optimistic extrapolation. Under fixed limited budgets, the respective thresholds ($1044, $1088, and $1169) are met at CD4 test costs of $5.15, $5.50 and $7.45.

## Discussion

Here we report the costs and economic outcomes associated with routine laboratory monitoring of ART using individual patient data from Uganda and Zimbabwe in the DART trial. Routine toxicity monitoring had no additional benefits on any adverse event outcome and was more costly than clinically driven toxicity monitoring. While effective, routine (12-weekly) laboratory CD4 count monitoring was more costly than monitoring without CD4 counts. Even ignoring routine toxicity monitoring, based on the ICERs derived for life-years or QALYs gained, the CD4 monitoring strategy used in DART is beyond the cost-effective threshold of <3x per capita GDP, which is the WHO benchmark used for assessing cost-effectiveness of interventions in low-income sub-Saharan African countries [30].

Since DART publication, three other randomised trials comparing different ways to monitor adults on ART in low and middle income countries have been presented or published: the HBAC trial in Uganda [11], [18], the PHPT-3 trial in Thailand [32] and the ANRS/ESTHER trial in Cameroon [33]. Although different in design, the two trials that included a clinical monitoring arm (Uganda, Cameroon) both show small but important benefits for CD4 laboratory monitoring and thus support the key observation from DART on which this cost-effectiveness analysis is predicated. Interestingly, HBAC and PHPT-3, the two studies that compared viral load with CD4 monitoring, have both failed to show any additional benefit on clinical outcome from virological monitoring, either in place of or additional to CD4 monitoring [32], [33].

According to our primary costing studies and models, we estimated that the current costs of CD4 tests need to drop to below USD$3.78 for 12-weekly CD4 monitoring to be cost-effective. Extrapolating over the longer-term under the fixed budget rule (instead of the arbitrary, conventional threshold used to approximate the value of a life year in optimal health or without disability [31]) that the option with most total benefits be selected suggested laboratory monitoring at unit costs of USD$5-7 would be cost-effective in a population starting ART with relatively severe immunodeficiency as in DART. In patients starting ART at higher CD4 counts, more CD4 counts would need to be done to identify failure, and so incremental cost effectiveness ratios would likely be higher, requiring lower unit cost of CD4 counts to achieve cost-effectiveness. Costs could reduce either through direct kit costs falling, or through efficiency gains for example with new technologies such as point-of-care (POC) tests. Such technology is now being developed and evaluated: if costs fall below $3–4 per test and the devices can be used at health-centre level then POC could become a very attractive option for routine ART efficacy monitoring [34]. Another option would be to perform CD4 monitoring less frequently than 12-weekly. However, as excess morbidity/mortality in CDM appeared to mainly be a consequence of later switch to second-line, less frequent CD4 monitoring could also be less effective, reducing cost savings.

The long-term economic value of routine CD4 monitoring is inversely related to the costs of second-line therapy: the lower the costs of second-line therapy, the greater the likelihood of CD4 monitoring being cost-effective. Of the three other trials of ART monitoring strategies,only the Uganda HBAC study so far has conducted cost-effectiveness analysis [18]. It reports an ICER of $174 per disability-adjusted-life-year (DALY) for CD4/clinical vs clinical monitoring alone of ART [18], an order of magnitude lower than the ICER reported here. This difference between our findings is initially perplexing, particularly as participants receiving CD4 or viral load monitoring also received full blood counts; of note, toxicity outcomes were not reported in HBAC [9]. However, the substantially increased cost of second-line versus first-line ART is likely to have contributed to their finding, as, in contrast to DART, more participants (n = 17) in the clinical monitoring alone group switched to second-line, compared to those receiving CD4 (n = 4) or CD4 and viral load (n = 7) monitoring.

DART and HBAC used different clinical thresholds to determine first-line failure and thus trigger switch to second-line ART. The most important difference is the inclusion of single WHO 3 events in the HBAC clinical criteria for switch (weight loss, unexplained fever, diarrhoea, oral candidiasis), which were not included in WHO guidelines [35], tend to be non-specific and are most associated with high CD4 counts/suppressed viral load [36]; in contrast clinical switch in DART was based on WHO 4 events alone (as per WHO guidelines [35]). Thus the cost differential between CD4 and clinical monitored groups will be exaggerated in HBAC by increased unnecessary premature switching in the clinical monitoring group, thus reducing the ICER. Given the influence of second-line costs on cost-effectiveness, and given that ART will be given life-long, it is unfortunate that HBAC was relatively underpowered to address this critical issue, with only 28 switches in total (as opposed to 675 in DART with more in the LCM arm from the second year of the trial).

Furthermore, the median of only 3 years of follow-up in HBAC, compared to 5 years in DART, inevitably has implications for the accuracy of longer term predictions based on observed data. Although our model did not adjust for the effect of past or ongoing opportunistic infections on the risk of death over and above that captured by current CD4 count, any resulting bias favouring clinical monitoring is likely to be reversed by the assumption of higher switch rates with CDM to second-line therapy at high CD4 counts throughout the period after DART.

To be eligible for DART, all potential participants underwent CD4 testing and needed to have CD4<200 cells/mm^3^ to enter the trial. Our analysis was therefore not able to consider the costs and benefits of CD4 testing for ART eligibility [4], [13], [16]. Initiating ART earlier, with higher CD4 counts, would make LCM less cost-effective, as fewer lives would potentially be saved by laboratory monitoring; subgroup analysis showed that the ICER among patients with CD4<100 cells/mm^3^ was 30% smaller than for the whole trial population (USD$6764 vs. USD$9621). As commented above, this would also lower the unit cost thresholds at which routine CD4 testing becomes cost-effective.

Our study is unique in its prospective valuation of patient preferences for health states in a sub-sample of DART patients. Interestingly, applying utility weights to health states in DART defined according to performance status and any ongoing WHO stage 3 or 4 events, as assessed by a doctor, showed that the estimated survival difference (0.101 life years) was associated with an almost equal gain in asymptomatic survival (0.097 life years).

DART used a simple and easy to interpret switch criterion of CD4<100 cells/mm^3^ which does not require longitudinal measurement of CD4 counts, or pre-ART counts. More complex failure criteria based on prior values (CD4 fall <pre-ART, >50% decline from peak) may increase the proportions switching to second-line without necessarily improving outcomes, thus reducing the likelihood of CD4 monitoring being cost-effective; these criteria are also more challenging to implement. Interestingly the HBAC study used persistently declining CD4 cell counts on two consecutive measurements to indicate treatment failure: this has been demonstrated to have the lowest sensitivity of the three failure criteria [37].

DART did not evaluate routine viral load testing. However, the difference between DART groups receiving and not receiving routine CD4 monitoring trial based was similar to one modelling study [15] and the HBAC randomised trial of laboratory monitoring in Uganda [9]. Both suggest limited additional benefits with viral load monitoring, highlighting the importance of defining the optimal threshold for virologic failure and submitting routine viral load monitoring to a definitive evaluation [38]. On the basis of a recent review [39] and the ICER of $5181 per DALY for viral load/CD4/clinical monitoring vs. CD4/clinical monitoring reported by the HBAC study [18], at current costs and with current technologies, there is less evidence of cost-effectiveness for viral load monitoring than there is for CD4 count monitoring.

A further limitation of the cost-effectiveness analysis presented here is that it has only considered the patients on ART, and in particular has not considered other possible impacts such as increased HIV transmission, including of drug resistant virus, from unsuppressed patients on ART or, in contrast, reduced transmission from having been able to put more patients on ART without providing routine laboratory monitoring for the same fixed budget. Modelling studies, when they have examined the evolution and transmission of drug resistance under a public health approach to ART, have been relatively reassuring [15], [40]. A modelling component within the Lablite project will further model these aspects.

Our findings have implications for public sector ART programmes in Africa, given the unmet demand for treatment, the limited availability of laboratory services and stagnant or declining international funding for health. Strengthening laboratory services is a priority: CD4 testing should focus on ART initiation; toxicity testing can be clinically guided rather than routine; access to quality-controlled diagnostic testing must be widened. With fixed and constrained budgets, relative to no ART provision [41], the number of lives/life-years saved with current costs and approaches is greater for a clinically-driven rather than laboratory monitoring strategy. Further, it is an equitable option as its lower cost allows more patients to access treatment and for follow up to be decentralised to health centres [42]. Resources could therefore better be allocated for untreated patients to start ART, and for laboratory diagnostic investigation of clinical events, rather than for routine laboratory monitoring [43]. The recently started LabLite project aims to demonstrate that such decentralised ART delivery (outside research clinics) is safe and effective in rural areas in Malawi, Uganda and Zimbabwe.

Results from the DART trial and the cost-effectiveness analysis also raise challenging issues about how to act on research findings when effectiveness is demonstrated but cost-effectiveness is not, as profound underlying tensions between patients, healthcare workers, funders and policy makers are exposed [44]. This paradox is a challenge for the public sector even in high-income countries (e.g. in the UK with the potential provision of very expensive cancer drugs with significant toxicity but also proven, limited benefits in the NHS), as resources universally are finite. It is even more difficult when interventions that are routinely provided in better resourced settings with higher GDPs, like CD4 testing, produce benefit but are not cost-effective in low-income settings. Particularly in resource-constrained settings, spending money on one healthcare component such as ART monitoring necessarily means fewer resources can be spent on other competing priorities (patients still in need of ART, bed nets to prevent malaria, vaccination programmes and so on) and we would argue our research helps policy makers to choose how to maximise health benefit.

In conclusion, the DART trial has clearly shown that routine laboratory monitoring of ART is not currently cost-effective in low-income African countries. There is no rationale for toxicity monitoring which does not affect outcomes and is costly. Routine CD4 monitoring has small but measurable benefits on survival; for it to be cost-effective the costs of second-line ART and CD4 testing need to fall substantially. Laboratories will remain important for quality of care especially for the diagnosis of intercurrent events on ART. Realising the survival benefits of CD4 monitoring widely in Africa is likely to be dependent on the development of a cheap, ideally point-of-care CD4 test. In the meantime, given competing priorities, HIV programmes in Africa may best spend limited resources on increasing access to first and second-line ART.

## Supporting Information

Supporting Information S1 **Modelling Utility Estimation in DART patients.**
(DOC)

Supporting Information S2 **Modelling the long term survival and costs of patients in DART.**
(DOC)
