# Supplementary material for: Cost Effectiveness Analysis of Clinically Driven versus Routine Laboratory Monitoring of Antiretroviral Therapy in Uganda and Zimbabwe
Source: PLoS One. 2012 Apr 24;7(4):e33672. doi: 10.1371/journal.pone.0033672 (PMC3335836; doi:10.1371/journal.pone.0033672)
Supplement: Supporting Information S1 — Modelling Utility Estimation in DART patients. (DOC) [file pone.0033672.s001.doc]

**Supporting Information S1**

**Utility Estimation in DART patients**

This appendix describes the estimation of utilities from a substudy of DART patients (n=275) at the Entebbe centre. Ethical approval for the HRQoL substudy was obtained from the Uganda Virus Research Institute Science and Ethics Committee, the Uganda National Council for Science and Technology and the Research Ethics Committee of the Liverpool School of Tropical Medicine.

Visual aids were used to describe health states and elicit patients’ utilities for WHO stages 2, 3 and 4 with the Time-Trade-Off (TTO) board; the psychometric performance of this tool has been validated in HIV infected Ugandans[1]. Individuals, interviewed face-to-face, in local language (Luganda) at enrolment (before ART), 6 and 12 months, expressed the amount of time they were willing to give up in order to be in a pre-defined improved health state rather than in WHO stage 2, 3 or 4.

Patients’ utilities for the three predetermined health states were analysed in a simultaneous, three equation (one for each predetermined state) linear item response model, including factors for age, sex, baseline CD4 counts, longitudinal performance scale assessments, WHO stage at recruitment and randomised allocation. The model accounted for the effect of intermittent missing data according to the framework proposed by Heckman[2] using maximum likelihood methods.

QALYs are presented using patients’ utilities, assuming that the utility of WHO stage 1, which was not measured in the HRQoL study, was equivalent to the best possible health state, implicitly set at 1. QALYs were also estimated using patients’ utilities multiplied by the value of the asymptomatic HIV state elicited from a representative sample of the general population, thereby re-scaling patients’ utilities to reflect societal preferences. The only identified study that has measured the asymptomatic HIV state using TTO in a sample from the general population reported a value of 0.81[3]. Therefore it is assumed that societal utilities include a 19% discount over the patients’.

**References**

1. Medina Lara A, Nyanzi-Wakholi B, Kasirye A, Munderi P, Watera C et al (2008) Utility assessment of HIV/AIDS related health states in HIV infected Ugandans. AIDS 22: S123–S130.
2. Heckman JJ (1979) Sample selection bias as a specification error. Econometrica 47: 153–161.
3. Staven K, Frolan SS, Hellum KB (2005) Comparison of preference-based utilities of the 15D, EQ-5D and SF-6D in patients with HIV/AIDS. Quality of Life Research 14: 971–980.
