# Supplementary material for: Cost Effectiveness Analysis of Clinically Driven versus Routine Laboratory Monitoring of Antiretroviral Therapy in Uganda and Zimbabwe
Source: PLoS One. 2012 Apr 24;7(4):e33672. doi: 10.1371/journal.pone.0033672 (PMC3335836; doi:10.1371/journal.pone.0033672)
Supplement: Supporting Information S2 — Modelling the long term survival and costs of patients in DART. (DOC) [file pone.0033672.s002.doc]

**Supporting Information S2**

**Modelling the long term survival and costs of patients in DART**

The experience of patients from the end of the 5^th^ year to year 25 after starting ART in DART was modelled as discrete CD4 count range transitions, including the transition to death, and assuming that patients continued their allocated laboratory monitoring strategy post-trial. A patient alive at the end of DART would be in one of three states defined by CD4 count, namely, <100, 100-200, and >200 cells/mm^3^. After every 12-week period thereafter, the patient would have moved to a different state, remained in the initial state or died from HIV related, treatment or unrelated causes. During the 12 weeks spent in a given state patients incur costs and accrue benefits associated with that particular state (for the purposes of the model, any movement back and forth between states and their associated changes in costs and benefits during the period is ignored as, by trial protocol, it is not observed) (Figure 1).

Figure 1 Markov transitions across CD4 count states and to death

Due to the expected differences in costs and possibly survival benefits, states are defined also by first- and second-line ART therapy. Figure 1 is simplified as for each one of the CD4 states there are in effect two states, one for first and another for second-line therapy. In principle this results in 7 possible transitions (3 CD4 states for first-line, 3 CD4 states for second-line and death) from any one first-line state. The three CD4 count states for second-line therapy have only four possible destinations, as it is not possible to make a transition back to first-line therapy from second-line therapy. No provision is made for salvage or third-line therapy.

***Estimation of Transition Probabilities***

Consistent with the finite number of discrete states in which an individual may be found at any given time, multinomial logit regression analysis was used to derive the transition probabilities. Separate equations, one for each CD4 state (<100, 100-200 and >200 cells/mm^3^) and line of therapy (i.e. first or second) at the start of the 12-weekly cycle of laboratory monitoring (as per research protocol for both groups[1]), were estimated as a function of factors.

Six regressions were estimated in total, one for each of the following initial states:

1. Low CD4 count (< 100 cells/mm^3^) and first-line therapy;
2. Low CD4 count and second-line therapy;
3. Medium CD4 count (100-200 cells/mm^3^) and first-line therapy;
4. Medium CD4 count and second-line therapy;
5. High CD4 count (>200 cells/mm^3^) and first line therapy and;
6. High CD4 count and second-line therapy.

For each of the regressions 1, 3, and 5 starting from a first-line regimen all 6 states were possible destinations, plus the death state. For regressions 2, 4 and 6 the possible destination states were the three states themselves plus the death state.

The multinomial specification may be interpreted as a competing risk model[2] where a given transition (e.g. from CD4 <100 to CD4 100-200 while on first-line) may be censored due to death, or other transition (e.g. to CD4 100-200 after switch to second-line). Therefore this approach was preferred to parametric survival regressions (e.g. Weibull) since handling competing risks in the latter approach involves complex distributional assumptions.

***Data and Methods***

The data set for each regression included all available CD4 counts and line of therapy observations for each initial state in question. Only adjacent, 12 week-apart CD4 measurements were included in the analysis. Similarly patients who died in the trial but had their last laboratory measurement taken more than 92 days before death were considered as censored (n=28/382). The outcome variable was state of destination.

Pre-ART age was included as a categorical factor (25 years or younger, 26-35, 46-55 and 55 and over; the band 36-45 was the reference). Since the risk of death and rate of switch varied over time in DART[1], the transition probabilities derived from the multinomial logit regressions controlled for age, sex, trial week number (weeks since ART initiation), randomised group, and, for regressions of second-line therapy states, week of initiation of second-line therapy (‘trial week number’ became ‘trial week number since start of second-line therapy’). In order to approximate mortality and switching to second-line therapy trends at the end of DART, the analysis dichotomised the trial week number variable into an indicator variable for 0 to 3 versus 3 to 6 years in DART (since median follow-up was 4.9 years, this resulted in an indicator for the first 3 versus the last two years in trial for the majority of patients); for regressions of transitions starting from second-line the same rule applied to the date of switch to second-line, while the number of weeks since start of second-line regimen was dichotomised into an indicator for 0 to 1 versus 1 plus years after switch. This was used to obtain predictions of transition probabilities conditional on the latter period of observation, as a way to make extrapolation on the basis of the latest observed pattern of death, switching and CD4 count transitions. Interactions between the indicator for the latter period and age, sex and group factors were included in the regressions.

Alternative estimation approaches were also explored that constrained the death risk to be the same within CD4 ranges and across lines of therapy as a way to test for the robustness of results. In these analyses the probability of death was modelled separately from CD4 transitions, which then became conditional on surviving the 12-weekly period of observation and for first line therapy states, had as additional factor the indicator of whether a transition was made to second-line therapy. This strategy required a separate analysis of switch to second-line therapy that was conditional on the patient being alive and thus observable over the 12-weekly period. Other sensitivity analyses were performed using an ordinal (logit or probit) specification for the random error distribution of the conditional CD4 transitions.

Extrapolations used mortality probabilities estimated from DART data. The analysis estimated the probability of death conditional on the latest CD4 by omitting deaths that were judged by the independent End-point Review Committee as unlikely to be related to HIV or treatment, and instead applying separately background mortality risks derived from counterfactual life tables for Uganda that exclude the effect of HIV/AIDS. These were derived from the WHO 1990 life tables[3] as they relate to a time when the condition had not yet become an epidemic. By preserving the relative age specific death risks for the life table of Uganda in 1990 and scaling up the death hazards by a common factor, the life tables were calibrated to the life expectancy at birth for Uganda in 2008 modelled by the US Census Bureau[4] under the hypothetical Without AIDS-Scenario (58.4 years for males and 62.3 years for females). This essentially calculates life tables without HIV-related death risks. Since counterfactual life expectancy at birth estimates without AIDS were not available for Zimbabwe, only the Ugandan calibrated life tables were used for extrapolation.

In order to account for the effects of uncertainty on results, simultaneous random sampling from the distribution of costs observed in DART, and parameters (transition probabilities) and pay-offs (resource quantities of nurse only, doctor visits and nights in hospital) in the model extrapolation was conducted in a probabilistic sensitivity analysis. DART observed costs were sampled from a normal distribution with mean and standard deviation as observed in the trial (from the Lin estimator[5] of mean and bootstrap estimate[6] of standard deviation of the mean difference); transition probabilities were sampled from the Dirichlet[7] distribution; pay-off quantities were derived from the gamma distribution with scale and shape parameters estimated using the method of moments. Sampling was implemented independently across the three groups of random variables and results were summarised in a cost-effectiveness acceptability curve.

Results were interpreted with reference to two alternative thresholds, representing mutually exclusive states of the world with regard to ability to pay for healthcare. The WHO cost-effectiveness threshold guidance of 3x per capita GDP in each country was used to represent the situation where treatment of all eligible patients under either monitoring option may be afforded. The alternative threshold, defined by the ICER of CDM relative to no ART treatment, was derived from survival experience without treatment in an observational cohort of HIV adult patients in Entebbe, Uganda[8]. Estimated discounted at 3% QALYs (9.61 over 25 years) in the DART CDM group were compared with the respective estimates for the Entebbe Cohort (EC), which were derived by applying overall, weighted average utilities in CDM for the duration of DART to the discounted mean survival estimates (1.43 life years, 1.18 QALYs over 25 years) from the area under the EC survival curve. Lacking further information, no public healthcare costs were assumed for patients without ART, and incremental costs of CDM were thus identical to its overall discounted costs over 25 years after the start of treatment.

***Results***

A total of 62,374 12-weekly observation periods were available for analysis, corresponding to 3316 DART participants. Divided by initial CD4 state, there were: 6973 (low CD4 and first line), 333 (low CD4 and second line), 14960 (medium CD4 and first line), 1091 (medium CD4 and second-line), 36004 (high CD4 and first line) and 3013 (high CD4 and second line).

Factors having coefficients with p values >0.05 in regressions including all factors were dropped from the final model used for predicting probabilities. Trial group was found to be a significant factor for transitions from first line at CD4≤100 and CD4>200. Different probabilities for male and female sex were estimated for transitions from all states except for the second-line and low CD4. This may have been the result of the small numbers available for that initial state. At least one age band was a significant predictor in all the starting state regressions.

The results of the multinomial equation estimates (not shown but available from authors upon request), implied the predicted transitions probabilities presented in Tables 1-4 below

**Table 1 Probabilities of transition between states over a 12-week period after 3 years in DART: LCM and female**

| **Initial State** | | **Final State** | | | | | | **Death** |
| --- | --- | --- | --- | --- | --- | --- | --- | --- |
|  |  | **First Line** | **Second Line** | **First Line** | **Second Line** | **First Line** | **Second Line** |  |
|  |  | **CD4>200** | | **CD4 100-199** | | **CD4<100** | |  |
| **First Line** | **CD4**  **≥200** | 0.9446 | 0.0010 | 0.0501 | 0.0001 | 0.0026 |  | 0.0016 |
| **Second Line^1^** |  |  | 0.9471 |  | 0.0436 |  | 0.0062 | 0.0031 |
| **First Line^2^** | **CD4**  **100-199** | 0.3241 | 0.0181 | 0.5743 | 0.0074 | 0.0741 | 0.0005 | 0.0015 |
| **Second Line** |  |  | 0.4360 |  | 0.5085 |  | 0.0469 | 0.0085 |
| **First Line^3^** | **CD4**  **<100** | 0.0392 | 0.1617 | 0.0964 | 0.2365 | 0.3909 | 0.0643 | 0.0110 |
| **Second Line^4^** |  |  | 0.3953 |  | 0.3255 |  | 0.2460 | 0.0331 |
| **Death** |  |  |  |  |  |  |  | 1 |

1 Transition probabilities from the Second Line and CD4>200 state varied at age >55: these were 0.0150, 0.0061, 0.0431, 0.9357, for the Death, CD4<100, CD4100-199, and CD4>200 destination states.

2 Transition probabilities from state First Line CD4 100-199 varied for Age 45-54: these were 0.0016, 0.0005, 0.0661, 0.0074, 0.5793, 0.0182, and 0.3269, for Death, second and first line CD4 <100, second and first line CD4 100-199, and CD4>=200 with second and first line, respectively.

3 Transition probabilities from first line CD4<100 state varied by age at 45-54, when the probabilities of Death, CD4<100 and First Line, CD4< 100 and Second Line, CD4 100-199 and First Line, CD4 100-199 and Second Line, CD4>200 and First Line and CD4>200 and Second Line were 0.0101, 0.0592, 0.3598, 0.2177, 0.1417, 0.1488, and 0.0627, respectively.

4 Transition probabilities from the second line CD4<100 state were allowed to vary by age: for those younger than 25, the probabilities to states of Death, CD$<100, CD$ 100-199, and CD4>=200 were 0.0548, 0.4069, 0.5383, and 0.0000, respectively; for the >=55 years group, the corresponding probabilities were 0.0484, 0.3599, 0.4761 and 0.1156.

**Table 2 Probabilities of transition between states over a 12-week period after 3 years in DART: LCM and m**ale

| **Initial State** | | **Final State** | | | | | | **Death** |
| --- | --- | --- | --- | --- | --- | --- | --- | --- |
|  |  | **First Line** | **Second Line** | **First Line** | **Second Line** | **First Line** | **Second Line** |  |
|  |  | **CD4>200** | | **CD4 100-199** | | **CD4<100** | |  |
| **First Line** | **CD4**  **≥200** | 0.9255 | 0.0010 | 0.0680 | 0.0001 | 0.0038 | 0 | 0.0016 |
| **Second Line^1^** |  |  | 0.9120 |  | 0.0790 |  | 0.0060 | 0.0030 |
| **First Line^2^** | **CD4 100-199** | 0.2755 | 0.0089 | 0.6171 | 0.0079 | 0.0884 | 0.0005 | 0.0017 |
| **Second Line** |  |  | 0.3587 |  | 0.5782 |  | 0.0534 | 0.0097 |
| **First Line^3^** | **CD4**  **<100** | 0.0255 | 0.1107 | 0.0832 | 0.2545 | 0.4206 | 0.0938 | 0.0118 |
| **Second Line^4^** |  |  | 0.3953 |  | 0.3255 |  | 0.2460 | 0.0331 |
| **Death** |  |  |  |  |  |  |  | 1 |

1 Transition probabilities from the Second Line and CD4>200 state varied at age >55: these were 0.0145, 0.0059, 0.0781, 0.9015, for the Death, CD4<100, CD4100-199, and CD4>200 destination states.

2 Transition probabilities from state First Line CD4 100-199 varied for Age 45-54: these were 0.0017, 0.0005, 0.0819, 0.0080, 0.6215, 0.0089, and 0.2774, for Death, second and first line CD4 <100, second and first line CD4 100-199, and CD4>=200 with second and first line, respectively.

3 Transition probabilities from first line CD4<100 state varied by age at 45-54: these were 0.0110 for Death; 0.0878 and 0.3936, for CD4<100, Second Line and First Line, respectively; 0.2382 and 0.1243 for CD4 100-199, Second Line and First Line; and, for CD4>200, 0.1036 and 0.0414, respectively.

4 Transition probabilities from the second line CD4<100 state were allowed to vary by age: for those younger than 25, the probabilities to states of Death, CD$<100, CD$ 100-199, and CD4>=200 were 0.0548, 0.4069, 0.5383, and 0.0000, respectively; for the >=55 years group, the corresponding probabilities were 0.0484, 0.3599, 0.4761 and 0.1156.

**Table 3 Probabilities of transition between states over a 12-week period after 3 years in DART: CDM and f**emale

| **Initial State** | | **Final State** | | | | | | **Death** |
| --- | --- | --- | --- | --- | --- | --- | --- | --- |
|  |  | **First Line** | **Second Line** | **First Line** | **Second Line** | **First Line** | **Second Line** |  |
|  |  | **CD4>200** | | **CD4 100-199** | | **CD4<100** | |  |
| **First Line** | **CD4**  **≥200** | 0.9383 | 0.0076 | 0.0498 | 0.0001 | 0.0026 |  | 0.0016 |
| **Second Line^1^** |  |  | 0.9471 |  | 0.0436 |  | 0.0062 | 0.0031 |
| **First Line^2^** | **CD4 100-199** | 0.3241 | 0.0181 | 0.5743 | 0.0074 | 0.0741 | 0.0005 | 0.0015 |
| **Second Line** |  |  | 0.4360 |  | 0.5085 |  | 0.0469 | 0.0085 |
| **First Line^3^** | **CD4**  **<100** | 0.0433 | 0.0460 | 0.1061 | 0.0632 | 0.6608 | 0.0619 | 0.0185 |
| **Second Line^4^** |  |  | 0.3953 |  | 0.3255 |  | 0.2460 | 0.0331 |
| **Death** |  |  |  |  |  |  |  | 1 |

1 Transition probabilities from the Second Line and CD4>200 state varied at age >55: these were 0.0150, 0.0061, 0.0431, 0.9357, for the Death, CD4<100, CD4100-199, and CD4>200 destination states.

2 Transition probabilities from state First Line CD4 100-199 varied for Age 45-54: these were 0.0016, 0.0005, 0.0661, 0.0074, 0.5793, 0.0182, and 0.3269, for Death, second and first line CD4 <100, second and first line CD4 100-199, and CD4>=200 with second and first line, respectively.

3 Transition probabilities from first line CD4<100 state varied by age at 45-54, when the probabilities of Death, CD4<100 and First Line, CD4< 100 and Second Line, CD4 100-199 and First Line, CD4 100-199 and Second Line, CD4>200 and First Line and CD4>200 and Second Line were 0.0169, 0.0566, 0.6033, 0.0577, 0.1547, 0.0420, and 0.0687, respectively.

4 Transition probabilities from the second line CD4<100 state were allowed to vary by age: for those younger than 25, the probabilities to states of Death, CD$<100, CD$ 100-199, and CD4>=200 were 0.0548, 0.4069, 0.5383, and 0.0000, respectively; for the >=55 years group, the corresponding probabilities were 0.0484, 0.3599, 0.4761 and 0.1156.

**Table 4 Probabilities of transition between states over a 12-week period after 3 years in DART: CDM and m**ale

| **Initial State** | | **Final State** | | | | | | **Death** |
| --- | --- | --- | --- | --- | --- | --- | --- | --- |
|  |  | **First Line** | **Second Line** | **First Line** | **Second Line** | **First Line** | **Second Line** |  |
|  |  | **CD4>200** | | **CD4 100-199** | | **CD4<100** | |  |
| **First Line** | **CD4**  **≥200** | 0.9195 | 0.0074 | 0.0676 | 0.0001 | 0.0038 |  | 0.0016 |
| **Second Line** |  |  | 0.9120 |  | 0.0790 |  | 0.0060 | 0.0030 |
| **First Line^3^** | **CD4 100-199** | 0.2755 | 0.0089 | 0.6171 | 0.0079 | 0.0884 | 0.0005 | 0.0017 |
| **Second Line** |  |  | 0.3587 |  | 0.5782 |  | 0.0534 | 0.0097 |
| **First Line^2^** | **CD4**  **<100** | 0.0270 | 0.0303 | 0.0880 | 0.0654 | 0.6833 | 0.0868 | 0.0192 |
| **Second Line^1^** |  |  | 0.3953 |  | 0.3255 |  | 0.2460 | 0.0331 |
| **Death** |  |  |  |  |  |  |  | 1 |

1 Transition probabilities from the Second Line and CD4>200 state varied at age >55: these were 0.0145, 0.0059, 0.0781, 0.9015, for the Death, CD4<100, CD4100-199, and CD4>200 destination states.

2 Transition probabilities from state First Line CD4 100-199 varied for Age 45-54: these were 0.0017, 0.0005, 0.0819, 0.0080, 0.6215, 0.0089, and 0.2774, for Death, second and first line CD4 <100, second and first line CD4 100-199, and CD4>=200 with second and first line, respectively.

3 Transition probabilities from first line CD4<100 state varied by age at 45-54: these were 0.0179 for Death; 0.0809 and 0.6371, for CD4<100, Second Line and First Line, respectively; 0.0610 and 0.1310 for CD4 100-199, Second Line and First Line; and, for CD4>200, 0.0282 and 0.0438, respectively.

4 Transition probabilities from the second line CD4<100 state were allowed to vary by age: for those younger than 25, the probabilities to states of Death, CD$<100, CD$ 100-199, and CD4>=200 were 0.0548, 0.4069, 0.5383, and 0.0000, respectively; for the >=55 years group, the corresponding probabilities were 0.0484, 0.3599, 0.4761 and 0.1156.

An inspection of the estimated transition probabilities reveals that second-line therapy is associated with greater risks of death. The risk of death is estimated to decrease with increasing CD4 count during first- and second-line therapy; however, second-line therapy in the high CD4 state (≥200) is estimated to have a higher risk of death than that with first-line therapy in the 100-199 CD4 state. This may be a reflection of the excess risk of death that occurs around switch to second-line therapy due to clinical events coinciding with the CD4 count rise seen on switching ART; or it may be a consequence of further non-recoverable immune loss with failure. Bias due to the selected nature of the small numbers on second line therapy may also be a factor. However, at CD4 count <100 the switch to second-line therapy represents an opportunity for the patient to reach a higher immunological status and thus reduce the death risk, which from the low to the high CD4 ranges is reduced ten-fold.

As expected, the probability of switching was substantially and significantly higher in the CD4<100 state in LCM than CDM after three years of DART. A key result is the estimated differential switch rate between trial groups at the high CD4 range. For example, by comparing Table 1 with Table 3, it may be seen that at CD4>200, although rates of switch are small in both groups, the probability of switching to second-line therapy in CDM is 57% higher than that in LCM (i.e. 0.0069 vs. 0.0044). In contrast, at the intermediate CD4 count range, no difference in switch rates were found between groups. These estimates, together with the continuous rise in CD4 counts during DART, explain the pattern (not shown) illustrated by fitting a flexible parametric survival curve[9] to the time to second-line therapy data in the trial, where the LCM probability of switch (hazard) curve rises steeply above that for CDM early on (after year one), but subsequently falls, crossing over at the 3.5 years point and remaining under it for the rest of the trial period.

Since by the time DART ended, the rising CD4 trend was still evident and the majority (~78%) of patients alive were still on first-line therapy, it is only natural to expect that the overall switching rate reverses as first-line therapy starts to fail and CD4 counts to decline. Although the effect of changing CD4 trends on the long term costs and benefits of the two monitoring options are not explored given the limited data (and the analysis favours the LCM option by assuming continued efficacy of first-line ART throughout the modelled period), the implications of ever healthier patients reaching the higher CD4 status are explored in two alternative scenarios for the analysis. In the optimistic scenario, the estimated transition probabilities including higher switch rates at high CD4 counts in CDM than LCM (and vice versa at low CD4 counts) are used to extrapolate the costs and benefits observed in DART to 20 years after its conclusion. In the conservative scenario, the extrapolation uses the transition probabilities modified so that both groups have the same probability of switching to second-line at the high CD4 count range, equal to the average estimate across groups in Tables 1 and 3, for Females, and 2 and 4, for Males.

***Pay-offs: Benefits***

Mean estimates of quality adjusted life quarters (12 weeks) by latest CD4 in DART are presented in Table 5 below. Two sets of estimates are presented according to whether patient utilities (for symptomatic states) are used to value time spent in health states (i.e. patient values) or patient valuations adjusted for the valuation of the asymptomatic HIV state by respondents from the general population are used (i.e. general population values). Health related quality of life is seen to decrease with lower CD4 values, although the decrease is of a higher magnitude for a fall in CD4 from the medium to low range than from the high to medium CD4 range. Group differences only occur for high and low CD4 values and are in every case minimal.

***Table 5 Utility values over 12-week by last CD4 and group***

|  | | **N** | **Utilities**  **- General Population Values**  **Mean (SD)** | **Utilities**  **- Patient Values**  **Mean (SD)** |
| --- | --- | --- | --- | --- |
| **LCM** | CD4>200 | 19664 | 0.613 (0.17) | 0.757 (0.21) |
|  | CD4 100-199 | 8119 | 0.606 (0.17) | 0.748 (0.22) |
|  | CD4<100 | 3111 | 0.548 (0.20) | 0.677 (0.25) |
| **CDM** | CD4>200 | 18519 | 0.608 (0.17) | 0.751 (0.21) |
|  | CD4 100-199 | 7762 | 0.606 (0.18) | 0.748 (0.22) |
|  | CD4<100 | 3979 | 0.541 (0.20) | 0.668 (0.25) |

***Pay-offs: Costs***

Event related cost pay-offs for the Markov model were similarly derived by analysing the respective cost category as a function of latest CD4 count state. Table 6 shows the estimates for each group. Costs tend to be higher the lower the CD4 count state. Use of hospital and doctor visits feature saliently as a cost driven by poor immunological status. It is also in terms of these costs that differences in event-related resource consumption between groups are noticeable; each group incurs excess costs relative to the other in one of the two cost categories: CDM is associated with lower doctor visit costs (at low CD4 counts) while it incurs higher hospital costs than LCM.

**Table 6 Event-related resource utilisation and costs over 12-week by last CD4 and g**roup

|  | | **Extra patient-initiated Lab tests: Haematology costs ($)** | | **Extra patient-initiated Lab tests: Biochemistry costs ($)** | **Nurse only visits** | | **Doctor Visits** | **Hospital nights** | |
| --- | --- | --- | --- | --- | --- | --- | --- | --- | --- |
|  |  | **N*** | **Mean (SD)** | **Mean**  **(SD)** | **N*** | **Mean**  **(SD)** | **Mean (SD)** | **N*** | **Mean (SD)** |
| **LCM** | **CD4**  **>200** | 14831 | 0.074  (1.91) | 0.821  (7.23) | 13566 | 0.006  (0.07) | 0.536  (0.77) | 13556 | 0.110  (1.18) |
|  | **CD4**  **100-199** | 3483 | 0.308 (4.59) | 1.655  (11.86) | 3294 | 0.006  (0.08) | 0.662  (0.94) | 3292 | 0.246  (2.41) |
|  | **CD4**  **<100** | 763 | 0.453 (4.56) | 3.271  (19.05) | 717 | 0.011  (0.10) | 1.322  (1.24) | 716 | 0.827  (5.00) |
| **CDM** | **CD4**  **>200** | 13784 | 0.119 (2.10) | 1.076  (10.28) | 12625 | 0.005  (0.07) | 0.535  (0.79) | 12621 | 0.135  (1.50) |
|  | **CD4**  **100-199** | 3117 | 0.239 (3.58) | 1.819  (11.88) | 2923 | 0.007  (0.10) | 0.605  (0.87) | 2923 | 0.281  (2.44) |
|  | **CD4**  **<100** | 1612 | 0.484 (4.74) | 5.764  (24.66) | 1474 | 0.005  (0.07) | 0.916  (1.16) | 1474 | 0.904  (4.66) |

* N: number of person – quarters with CD4 match in the relevant range.

**Extrapolation Results**

Outcomes were extrapolated by applying predicted transition probabilities for the last two years of DART. In the resulting optimistic scenario, with an overall higher switching rate with CDM (as the switching differential at high CD4s, where most patients spend time outweighs the offsetting differential at low CD4), this group would have a greater proportion of surviving patients on second-line by the third year after trial closure (the eighth after ART initiation, see Figure 1 in main text, top panel, left graph); that is, during DART and initially after trial end, more CDM patients remain on first-line but from three years subsequently and thereafter more LCM patients would remain on first line. As mortality risks are higher on second-line than first-line for all CD4 strata and in LCM and CDM, the mortality difference between LCM and CDM (with more patients with high CD4s switching to second-line) is maintained. In the conservative scenario, where groups have the same switching rates at high CD4 counts after DART, switch rates converge and LCM keeps accumulating additional patient time on second-line while its survival advantage (see below and Figure 1 in main text, middle panel, right graph in main text) declines because relatively few LCM participants enter the lowest CD4 state where mortality is high and can benefit from switching yet more patients are exposed to higher death risks on second line (see discussion of transition matrices in methods of this Appendix). These trends will have different implications for long-term cost-effectiveness.

The extrapolated survival curves have a logistic shape that reflects the excess mortality associated with older age (see Figure 1 in main text, middle panel). In the conservative scenario, about 60% of DART patients would have died by 25 years and the two survival curves converged (Figure 1, middle panel, right graph). Convergence occurs because the difference in survival is due entirely to more switching to second line in LCM in the low CD4 count range; over time ever diminishing numbers remain in such range and thus can benefit from laboratory monitoring. These results may be summarised intuitively by representing the uncertainty scenario where most of those patients who benefit from early switch by having access to LCM go on to experience death at some point within the 20 years after DART; benefits have limited duration.

In contrast, in the optimistic scenario, the survival gap is maintained after DART by the higher switch rate in CDM and an excess death risk associated with switching to second line therapy in the high CD4 count range in both groups (see Figure 1 in main text, middle panel, left graph). Thus, while survival gains from switching at low (<100 cell/mm^3^) contribute proportionally less to overall survival as more patients move to higher CD4 count ranges, death risks independent of latest CD4 (e.g. possibly related to a higher prevalence of history of opportunistic infection in CDM than in LCM at high CD4 counts) become more important determinants of survival differences.

The evolution of the ICER over the analytic time horizon is shown in Figure 1, bottom panel, where limited Laboratory and Clinical Monitoring is depicted under three cost conditions: a) CD4 cost observed in DART ($14.46, weighted average across centres); b) hypothetical, CD4 test ($3.78) and c) CD4 test and lowest observed second-line costs ($763; see Table 1 in results). The scenarios employ QALYs derived from utility valuations by patients, as opposed to those adjusted for valuation by the general population and discounts costs and benefits by 3% annually. The optimistic scenario is characterised by the downward evolution of the ICER after the trial (see Figure 1 in main text, bottom panel, left). This is driven by increasing second line drug cost savings from declining switch rates to second line therapy (which reduce the growth rate of the numerator of the ICER), alongside a constant rate of accrual of survival benefits (and constant growth rate in the denominator of the ICER) due to lasting benefits of avoiding exposure to clinical events by switching early at low CD4 counts with CD4 monitoring.

By accumulating a survival advantage, and avoiding expensive, inefficient use of second-line therapy in high CD4 count ranges, CD4 monitoring becomes cost-effective with a test cost of $3.78. Given low second-line therapy costs, the cost-effective threshold is found at a test cost of $6.75. If the budget is insufficient to treat all eligible patients under LCM monitoring, more lives, life-years may be saved and more morbidity avoided by the less expensive and less effective option as it may reach sufficiently more patients to compensate for its reduced effectiveness. This can be assessed by comparing the ICER of CD4 monitoring with that of CDM relative to the case of no ART being available, estimated as $1100 for the whole sample of DART (each centre would have a different ratio as their costs of CDM differ). The ICER would be equal to this threshold at CD4 test costs of $5.81.

In the conservative scenario the ICERs tend to increase as the analysis extends beyond trial closure. This is driven by a widening of the gap in second-line therapy use between groups (see Figure 1 in main text, bottom panel, right). This driver is stronger the lower the cost per CD4 count that applies, although the effect would be dampened in the case of policy making having a stronger preference for early outcomes (i.e. a higher discount rate). In order for the ICER curve to remain under the $1200 cost per QALY gained with limited CD4 monitoring (CD4 from the second year on ART only) and a low cost of second-line ART, the cost per CD4 count test would have to be $0.95. The same cost would have to fall further under a fixed, limited budget case, up to $0.15, in order for laboratory monitoring to be cost-effective. When utilities adjusted for general population valuations are used reducing the cost of CD4 count monitoring to zero is not sufficient to make it cost-effective.

Contrary to the optimistic scenario, results in the conservative scenario are found to be robust to the cost of second line-therapy, as the extra consumption of second-line treatment by participants under laboratory monitoring in the first 8 years of treatment would be offset by the opposite situation starting 3 years after DART, which is more heavily discounted as lies farther into the future. Similar results to the conservative scenario were obtained for estimates derived from statistical models that estimated death risks as a function of CD4 count state only, so that the probability of death was conditionally independent from first- or second-line ART treatment. Results were also robust to using an ordinal logit or probit model specification instead of the multinomial form for the analysis of CD4 count range transitions.

The effect of uncertainty was investigated by randomly sampling from the distribution of costs observed in DART, model parameters (transition probabilities) and pay-offs (resource quantities of nurse only, doctor visits and hospital nights) for probabilistic sensitivity analysis (Figure 2a, main text). The figure depicts the observed condition (a): CD4 test, $14.46; and observed second-line costs, $968 for both the conservative and the optimistic scenarios. The probability of cost-effectiveness is low by conventional standards (depicted), and lowers still if subject to a limited budget constraint (not shown).

Since the probabilistic sensitivity analysis was trivial for the conservative scenario, further analysis by centre is only presented for the optimistic scenario (Figure 2b, main text). The DART trial centre in Entebbe had a 3.5% probability of being cost-effective at the current costs per CD4 count of $15.79 given the 2008 threshold of cost-effectiveness of $1364 in Uganda. However, with a lower CD4 count cost of $10.07 in Kampala, there was a 30% probability of being cost-effective. In Harare, with CD4 cost of $18.82 and the 2008 threshold of cost-effectiveness of $804, there was zero probability cost-effectiveness. The cost-effectiveness threshold is achieved at test costs of $7.87, $7.43, and $3.39, respectively, in the three centres. Under fixed, limited budgets the respective thresholds ($1044, $1088, and $1169) are met at $5.15, $5.50 and $7.45.

**REFERENCES**

1. DART Trial Team (2010) Routine versus clinically driven laboratory monitoring of HIV antiretroviral therapy in Africa (DART): a randomised non-inferiority trial. Lancet 375: 23–31.

2. Steele F, Goldstein H, Browne W (2004) [A general multilevel multistate competing risks model for event history data, with an application to a study of contraceptive use dynamics](http://www.cmm.bristol.ac.uk/team/HG_Personal/Full Publications - download/2004/multilevel-multistate-event-history-model.pdf). Statistical Modelling 4:145–159.

3. WHO Life tables 1990. Available: <http://apps.who.int/whosis/database/life_tables/life_tables.cfm>. Accessed 2010 June 25.

4. US Census Bureau Country Profiles. Available: <http://www.census.gov/ipc/www/hiv/hivctry.html>). Accessed 2010 June 25.

5. Lin DY (2000) Linear regression analysis of censored medical costs. Biostatistics 1: 35–47.

6. Efron B, Tibshirani R (19930) An Introduction to the Bootstrap. New York: Chapman and Hall.

7. Briggs A, Ades A, Price NJ (2003) Probabilistic sensitivity analysis for decision trees with multiple branches: use of the dirichlet distribution in a Bayesian framework. Medical Decision Making 23: 341–350.

8. Munderi P, Watera C, Nakiyingi J, Gilks CF, Walker S et al (2006) Survival and causes of death, 2 years after introduction of antiretroviral therapy in Africa: a historical cohort comparison in Entebbe, Uganda. XVI International AIDS Conference. Toronto, Canada; Aug 13–18, 2006. Abstract THLB0208.

9. Royston P, Parmar MKB (2002) Flexible proportional-hazards and proportional-odds models for censored survival data, with application to prognostic modelling and estimation of treatment effects. Statistics in Medicine 21: 2175–2197.
